# Human Papillomavirus Positivity and Genotype Distribution Among Korean Females: A Large-Scale Laboratory-Based Analysis

**DOI:** 10.3390/jcm15176853

**Published:** 2026-09-04

**Authors:** Jae Hyun Baik, Mi Mi Oh, Seon Beom Jo, Du Geon Moon

**Affiliations:** Department of Urology, Korea University Guro Hospital, Korea University College of Medicine, 148 Gurodong-ro, Guro-gu, Seoul 08308, Republic of Korea

**Keywords:** human papillomavirus, genotype distribution, HPV positivity, co-infection, 9-valent vaccine, Korea

## Abstract

**Background/Objectives**: Human papillomavirus (HPV) genotype distribution is an important epidemiologic indicator for guiding public health surveillance, yet large-scale, laboratory-based data among Korean females remain limited, particularly following the incorporation of HPV vaccination into the National Immunization Program in 2016. This study evaluated HPV positivity and genotype distribution among clinically tested Korean females using a large-scale laboratory-based dataset. **Methods**: A retrospective analysis was conducted on 530,808 HPV genotyping results from Korean females tested between 2014 and 2022 using the Anyplex™ II HPV 28 Detection system (Seegene, Seoul, Republic of Korea). Specimens were classified as high-risk (HR), low-risk (LR), or multiple-type infections, and the proportion of specimens containing at least one HR genotype not included in the 9-valent vaccine was determined. **Results**: The overall HPV positivity rate was 44.4%, with a U-shaped age distribution. HPV 52 was the most frequently detected genotype (7.2%), followed by HPV 53 (6.3%) and HPV 58 (5.1%). Multiple-type infections were identified in 45.4% of HPV-positive specimens, and 24.4% of specimens harbored at least one HR genotype not included in the 9-valent vaccine. **Conclusions**: This large-scale laboratory-based analysis provides updated epidemiologic data on HPV positivity and genotype distribution among clinically tested Korean females. As this cohort reflects clinically tested rather than population-based specimens, these findings should be interpreted as descriptive observations that support continued surveillance of circulating HPV genotypes.

## 1. Introduction

Human papillomavirus (HPV) is one of the most common sexually transmitted infections worldwide [1,2]. Decades of clinical evidence have confirmed that persistent infection with high-risk strains, particularly carcinogenic types 16 and 18, is the central cause of cervical carcinogenesis [2]. These high-risk HPV infections extend beyond the cervix; they are also linked to a substantial proportion of other anogenital and oropharyngeal malignancies, including vaginal, anal, and vulvar cancers [3]. To address the profound public health impact of these cancers, prophylactic HPV vaccination has emerged as a key strategy for primary prevention.

The 9-valent HPV vaccine targets HPV types 6, 11, 16, 18, 31, 33, 45, 52, and 58 and is estimated to prevent nearly 90% of cervical cancer cases globally [4,5]. Population-based studies have demonstrated substantial reductions in vaccine-type HPV infections and related cervical lesions following the implementation of national vaccination programs [6]. However, genotype distribution varies across geographic regions and populations [7,8]. Since currently available vaccines target a defined set of HPV types [3], understanding regional variation in HPV genotype distribution remains important for characterizing HPV epidemiology and supporting ongoing surveillance efforts. Furthermore, following the introduction of HPV vaccination, circulating HPV genotype distributions may shift over time. Continued surveillance is therefore needed to better understand contemporary HPV epidemiology.

Within Korea, national HPV immunization programs have targeted adolescent girls [9]. Prior Korean investigations have reported HPV genotype composition in selected cohorts and screening-based populations, including regional university samples and lesion-associated screening studies [10,11,12]. However, many of these studies were based on relatively smaller cohorts or shorter study periods. Building on this body of work, updated large-scale, laboratory-based data spanning multiple years may help provide a more comprehensive and contemporary characterization of circulating HPV genotypes in Korea. Since the incorporation of HPV vaccination into the National Immunization Program (NIP) in Korea in 2016 [9], updated large-scale data describing circulating HPV genotypes are needed to better characterize contemporary HPV epidemiology.

In this study, we evaluated HPV positivity, genotype distribution, and infection patterns among clinically tested Korean females using a large-scale laboratory-based dataset and standardized testing methods. The scale of this dataset allowed detailed analyses of high-risk, low-risk, and multiple-type infections across age groups. However, anatomical sampling site was not recorded, so these findings reflect HPV surveillance among clinically tested females broadly, rather than site-specific cervical epidemiology.

## 2. Materials and Methods

### 2.1. Main Study Population

From March 2014 through March 2022, a total of 542,825 female specimens were submitted for HPV detection and genotyping, either through clinician referral or self-requested testing. The dataset reflects a real-world collection of specimens submitted from routine clinical practice across the country rather than a structured population-based screening program. Information regarding anatomical sampling sites and collection devices was not available, precluding site-specific analyses. Therefore, the findings should be interpreted as reflecting a clinically tested cohort rather than a general population. Because unique patient identifiers were unavailable and analyses were based on laboratory accession numbers, repeated testing of the same individual during the study period could not be ruled out. Accordingly, the dataset should be considered specimen-based rather than patient-based. Of the 542,825 specimens initially identified, 11,995 were excluded due to missing age information and 22 were excluded because the individual was aged <10 years, yielding a final analytic sample of 530,808 specimens for all analyses.

### 2.2. HPV DNA Testing

HPV detection and genotyping were performed using the Anyplex™ II HPV 28 system (Seegene, Seoul, Republic of Korea), which detects 28 HPV genotypes (19 high-risk (HR) and 9 low-risk (LR) types). DNA extraction and amplification were performed according to the manufacturer’s instructions. This real-time polymerase chain reaction (PCR) assay utilizes dual priming oligonucleotides (DPO™) and tagging oligonucleotide cleavage and extension (TOCE™) technology for melting curve analysis. During amplification, the HPV L1 gene and human β-globin were co-amplified, and fluorescence signals were continuously monitored as temperature increased. Human β-globin served as an internal control to verify specimen adequacy. Specimens failing β-globin amplification were deemed invalid according to the manufacturer’s criteria and were excluded from analysis prior to data provision by the testing laboratory; accordingly, the 542,825 specimens described in Section 2.1 already reflect only valid, β-globin-positive specimens. As this exclusion step was performed internally by the testing laboratory prior to data provision, the exact number of β-globin-negative specimens excluded was not available to the study team and could not be retrospectively retrieved. HPV genotypes were classified according to the risk categories of the Anyplex™ II HPV 28 assay, which are based on IARC criteria.

Co-infection was defined as the detection of two or more HPV genotypes in a single specimen. Co-infection categories (≥2 HR genotypes, ≥2 LR genotypes) were further analyzed according to the number and risk classification of detected HPV genotypes. A subset of specimens harbored both ≥2 HR and ≥2 LR genotypes simultaneously and were therefore counted in both categories, whereas specimens with exactly one HR and one LR genotype were not captured by either category. As a result, the sum of category-specific counts does not correspond exactly to the total number of specimens with ≥2 detected genotypes. A simple aggregate count of co-infecting genotypes would obscure the risk-category composition of each specimen. Combining high-risk and low-risk genotypes into a single unstratified “mixed” category can underestimate the true burden of high-risk HPV infection; for example, a specimen co-infected with a low-risk and a high-risk genotype would be classified merely as “mixed” rather than recognized as a high-risk infection, understating the proportion of high-risk-positive specimens in the cohort [13]. This distinction is clinically relevant given that high-risk genotypes are associated with cervical carcinogenesis [2], whereas low-risk genotypes, particularly HPV6 and HPV11, are responsible for approximately 90% of genital warts [14]. The risk-stratified classification used here was therefore adopted to preserve this distinction.

### 2.3. Statistical Analysis

Statistical analyses were performed using IBM SPSS Statistics version 22 (IBM Corp., Armonk, NY, USA). Categorical variables are presented as numbers and percentages. Differences in overall HPV positivity, high-risk HPV positivity, and co-infection rates across age groups were evaluated using the chi-square test. Given the specimen-based nature of the dataset, these comparisons were intended to provide descriptive information regarding age-related patterns. As no unique patient identifiers were available, deduplication of repeated specimens from the same individual was not feasible, and reported *p*-values should therefore be interpreted with caution given that the independence assumption underlying the chi-square test cannot be guaranteed in this specimen-based dataset. All statistical tests were two-sided, and a *p*-value of <0.05 was considered statistically significant. Age was categorized into seven predefined groups (10–19, 20–29, 30–39, 40–49, 50–59, 60–69, and ≥70 years) for age-based comparisons.

### 2.4. Ethics

This study was approved by the Institutional Review Board of the Seegene Medical Foundation (IRB approval number: SMF-IRB-2022-017). The requirement for informed consent was waived due to the use of de-identified data.

## 3. Results

### 3.1. Overall HPV Detection and Study Population Characteristics

**Figure 1:**
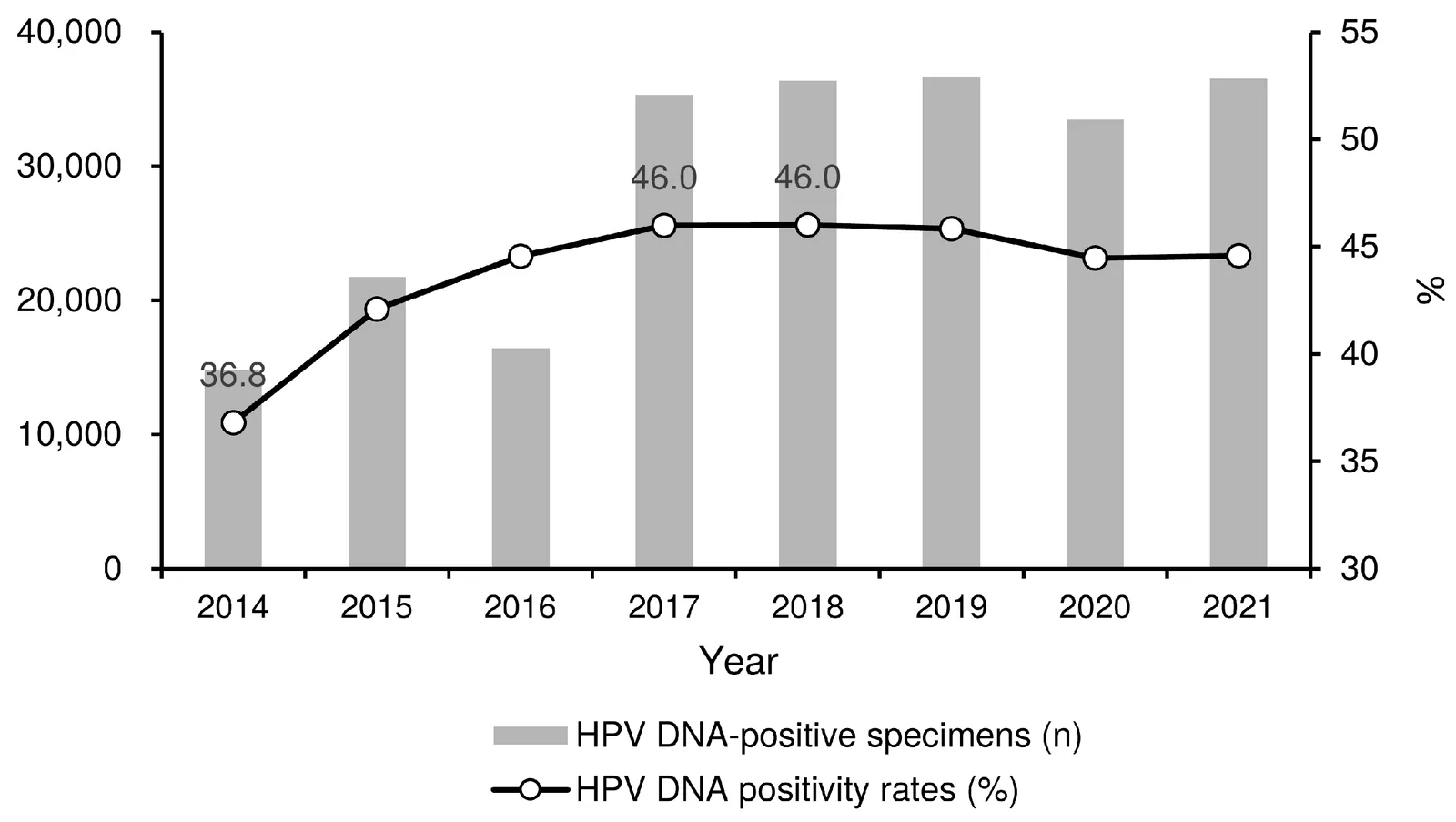
Annual number of HPV DNA-positive specimens and positivity rates among Korean females (2014–2021). Data from 2022 (*n* = 8849) were excluded from this figure because they represented incomplete-year observations; the full 2014–2022 dataset is described in Section 2.1. The secondary y-axis (positivity rate, %) does not start at zero.

Among 542,825 submitted female specimens, 530,808 with available age information were included in the final analysis. Of these, 235,626 were HPV DNA-positive, corresponding to an overall positivity rate of 44.4%. Annual testing volumes varied across the study period. Because 2022 data represented only partial-year observations (*n* = 8849), this year was excluded from the annual comparison shown in Figure 1. Among the remaining years (2014–2021), annual HPV positivity rates ranged from 36.8% to 46.0%, with an initial increase through 2017 followed by relatively stable rates between 2017 and 2021 (Figure 1).

### 3.2. Distribution of HPV Genotypes by Risk Category

HPV 52 was the most frequently detected genotype (7.2%), followed by HPV 53 (6.3%) and HPV 58 (5.1%). Among high-risk (HR) genotypes, HPV 52, 53, 58, 68 (4.9%), 16 (4.3%), and 51 (4.1%) were most frequently detected. Among low-risk (LR) genotypes, HPV 54 (5.4%) and HPV 70 (4.0%) were the most frequently detected, while other LR types were observed at lower frequencies (Table 1).

### 3.3. Patterns of Multiple HPV Infections

During the study period, single-genotype infection accounted for 21.4% to 25.4% of all tested specimens annually. In total, 128,769 cases (54.6% of HPV-positive specimens) were classified as single-type infections. Double infection accounted for 8.8–11.6% of tested specimens each year and represented the next largest infection category (Table 2).

Overall, 106,857 specimens (45.4% of HPV-positive cases) demonstrated infection with multiple HPV genotypes. Within this group, 66,553 (62.3% of co-infected cases) involved two or more HR genotypes, and 26,184 (24.5% of co-infected cases) involved two or more LR genotypes. A subset of specimens harbored both ≥2 HR and ≥2 LR genotypes simultaneously and were therefore counted in both categories, whereas specimens with exactly one HR and one LR genotype were not captured by either category; as a result, these counts do not sum exactly to the total number of specimens with multiple HPV infections. Concurrent infection with both HPV 16 and HPV 18 was documented in 938 cases (0.4% of HPV-positive specimens) (Table 3).

### 3.4. Age-Stratified HPV Positivity

Age-specific HPV positivity followed a distinct U-shaped distribution across age groups (*p* < 0.001, interpreted descriptively given the specimen-based nature of the dataset). The highest positivity was observed in adolescents (65.6%), followed by women in their 20s (59.3%). Positivity declined among women in their 30s and reached its lowest level in their 40s (36.5%), after which it increased in older age groups, reaching 51.1% among women aged ≥70 years.

HR-HPV and LR-HPV positivity showed similar age-related patterns, with higher positivity observed in younger age groups and lower positivity in middle-aged women. Both HR-HPV and LR-HPV positivity varied across age groups (*p* < 0.001, interpreted descriptively).

**Figure 2:**
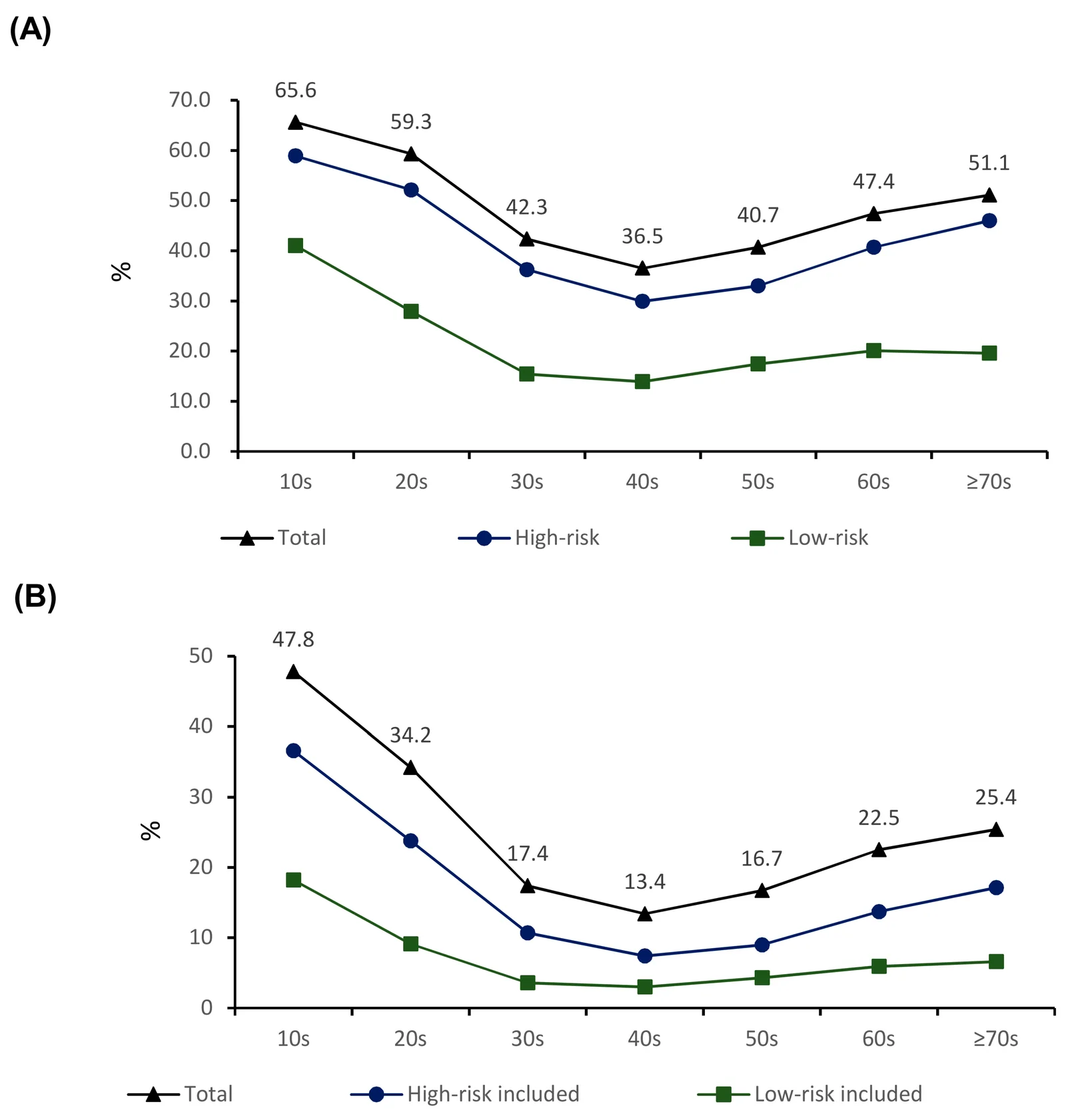
Age-specific HPV positivity according to infection type. (**A**) Positivity rates of total HPV, high-risk HPV, and low-risk HPV by age group. (**B**) Positivity rates of multiple HPV infections by age group and risk category; “high-risk included” and “low-risk included” denote multiple-type infections in which at least one high-risk or low-risk genotype, respectively, was detected (categories not mutually exclusive). The numbers of specimens included in each age group are provided in Supplementary Table S1. The underlying multiplicity data shown in panel (**B**) are provided in Supplementary Table S2.

Co-infection patterns also varied across age groups (*p* < 0.001, interpreted descriptively). Infection with two or more HPV genotypes was most frequent in adolescents and declined with advancing age. Multiple HR-type and multiple LR-type co-infections followed similar age-related distributions, with modest increases observed in older age groups (Figure 2). Age-group denominators are provided in Supplementary Table S1, and the corresponding multiplicity data are provided in Supplementary Table S2.

### 3.5. Positivity of Non-9-Valent High-Risk Genotypes

When the analysis was restricted to HR genotypes not included in the 9-valent HPV vaccine, positivity rates of individual non-9-valent HR genotypes ranged from 0.2% to 6.3%. Among the non-9-valent HR genotypes, the most frequently detected types were HPV 53 (6.3%), HPV 68 (4.9%), HPV 51 (4.1%), HPV 56 (3.8%), and HPV 39 (3.8%) (Table 4).

In total, 24.4% of specimens harbored at least one non-9-valent HR genotype. The proportion of specimens positive for at least one non-9-valent HR genotype varied across the study period, ranging from 19.3% in 2014 to 27.1% in 2022 (Supplementary Table S3). Data for 2022 represents a partial year of specimen collection.

## 4. Discussion

In this laboratory-based analysis of 530,808 female specimens, HPV DNA was detected in 44.4% of samples tested. Positivity increased from 36.8% in 2014 to approximately 46% by 2017 and remained relatively stable between 2017 and 2021. 2022 was excluded from this annual comparison because it represented incomplete-year data (*n* = 8849). Previous Korean screening-based studies have reported HPV positivity rates ranging from approximately 16% to 20% among women, including Ouh et al. [11] (*n* = 18,170, 2014–2016) and Kim et al. [15] (*n* = 7014, 2008–2010, overall positivity 19.7%). However, direct comparisons should be interpreted with caution because the present dataset was derived from clinically submitted specimens rather than a structured population-based screening cohort.

Among the 235,626 HPV-positive specimens, HPV52 was the most frequently detected genotype (16.2%), followed by HPV53 (14.1%) and HPV58 (11.4%). The predominance of HPV 52 and HPV 58 in this cohort is consistent with prior reports from East Asian populations, where these genotypes have been documented at relatively higher frequencies compared with Western regions [16]. Similar regional differences in genotype have been reported in large-scale global meta-analyses [8,17]. The genotype distribution observed in the present study was consistent with previous Korean data, including Lee et al. [18] (*n* = 60,775), who reported HPV16, 52, and 58 as common HR genotypes, Ouh et al. [11], who identified HPV53, 58, and 52 as dominant HR types, and So et al. [12] (*n* = 968), who similarly identified HPV53, 52, and 58 as the most prevalent HR genotypes, while HPV54 and HPV70 were frequently reported as the leading LR types [11,12]. More recently, Kim et al. [19] (*n* = 16,669) similarly reported multi-type infection rates of 15.3%, with double-, triple-, and quadruple-or-more-type infections comprising 8.6%, 3.8%, and 2.9%, respectively, and observed an age-related pattern similarly characterized by a decline through subjects in their 40s followed by a rebound in older age groups.

Taken together, these prior Korean studies indicate that the genotype and age-related patterns observed in the present study are confirmatory rather than novel.

However, genotype frequency should not be interpreted as a direct measure of carcinogenic risk. HPV 53 has been reported to be relatively common in population-based studies despite limited evidence supporting a major role in cervical carcinogenesis [20]. Therefore, genotype frequency should be interpreted separately from genotype-specific cancer risk.

Unlike patient-level longitudinal studies such as Song et al. [21], which linked individual vaccination history with HPV persistence and CIN2+ outcomes, the present cross-sectional, specimen-based dataset cannot address vaccine effectiveness directly; this distinction further underscores that the defensible contribution of the present study lies in its scale and laboratory-based genotype surveillance, rather than vaccine-effect inference.

Multiple-type infection was identified in 45.4% of HPV-positive specimens. Of these, 66,553 (62.3% of co-infected cases) involved multiple HR genotypes, while 26,184 (24.5%) involved multiple LR genotypes. This proportion is broadly comparable to that reported in previous cohort and population-based studies. In the Costa Rica Vaccine Trial cohort, multiple-type infections were observed in approximately 30% to over 50% of HPV-positive women depending on disease category [22]. Similarly, a population-based study from Uzbekistan reported multiple infections in approximately 20% of HPV-positive women [23], while longitudinal data from Rousseau et al. demonstrated co-infection rates of around 20% across repeated visits [24]. The variability across studies likely reflects differences in age structure, underlying disease severity, and detection methodologies. The higher multiplicity observed in the present cohort, similar to the elevated overall positivity rate, may also reflect the clinically enriched nature of this specimen population rather than a true population-level phenomenon. Nevertheless, our findings are broadly consistent with prior large-scale cohorts, supporting the substantial contribution of multiple-type infections in HPV epidemiology. Previous longitudinal studies have reported associations between multiple-type infection and differences in viral persistence dynamics [24,25,26], although the clinical implications of concurrent HR infections remain incompletely defined. These observations suggest that genotype distribution and co-infection patterns remain important components of HPV surveillance. Concurrent detection of two or more genotypes among HPV52, HPV53, and HPV58—the three most frequently detected HR genotypes in this dataset—was observed in 10,242 specimens (1.9% of all tested specimens), reflecting the regional predominance of these genotypes in the present cohort.

Age-specific analyses showed higher HPV positivity in adolescents and young adults, followed by a decline in middle age and a secondary increase in older women. Comparable patterns, including a secondary rise in older age groups, have been reported in population-based and international analyses [17,22,27]. A similar U-shaped age distribution was reported by Lee et al. [18] among 60,775 Korean women tested between 2006 and 2011. While the precise mechanisms underlying this pattern remain incompletely understood, prior studies have emphasized age-related differences in HPV acquisition and persistence, as well as uncertainties surrounding viral clearance and latent infection [28,29,30]. However, because the present study was based on clinically submitted specimens rather than a population-based screening cohort, differences in testing indications across age groups may have contributed to the observed age-specific positivity patterns.

Although HPV types 16 and 18 account for the majority of cervical cancer cases worldwide [3], and the 9-valent vaccine extends protection to five additional oncogenic types [4], not all carcinogenic genotypes are included. In this study, 24.4% of specimens contained at least one HR genotype not included in the 9-valent vaccine. This proportion was not evenly distributed across age groups, ranging from 18.5% in the 40s to 47.2% in the 10s, following a U-shaped pattern broadly consistent with overall HPV positivity (Supplementary Table S4).

As noted above, the anatomical sampling site was unavailable for this dataset; accordingly, the genotype-specific analyses below should be interpreted as laboratory surveillance rather than cervical-disease-specific epidemiology. To further characterize the carcinogenic evidence underlying this estimate, non-9-valent HR genotypes were additionally stratified by IARC classification (Table 4). Genotypes with the strongest evidence of carcinogenicity (IARC Group 1: HPV35, 39, 51, 56, 59) accounted for 13.7% of specimens, increasing to 17.4% when Group 2A (HPV68, probably carcinogenic) was included. The remaining difference between this value and the overall 24.4% assay-defined estimate—corresponding to Group 2B genotypes (HPV26, 53, 66, 73, 82, possibly carcinogenic; 10.6%)—is largely attributable to HPV53, the second most frequently detected genotype in this cohort. As HPV53 carries comparatively weaker evidence of carcinogenicity, the assay-defined 24.4% estimate should be interpreted with caution, as it may overstate the burden of genotypes with confirmed oncogenic potential. Nevertheless, even when restricted to Group 1 genotypes alone, 13.7% of specimens harbored at least one non-9-valent HR genotype of confirmed carcinogenicity.

As a female counterpart to a previously reported male cohort analyzed using the same testing platform, the present study similarly identified HPV 51, 39, 53, and 66 (the latter two also identified as major Group 2B contributors above) among the most frequently detected non-9-valent HR genotypes [31], suggesting shared genotype circulation patterns across sexes within this cohort. This study also shares the same laboratory setting and analytic framework as the companion male analysis, though the present analysis additionally incorporates IARC-based carcinogenicity stratification not included in that companion dataset.

To facilitate interpretation of year-to-year variation, we additionally provide age-by-calendar year data in Supplementary Table S3. Although the overall proportion of specimens with ≥1 assay-defined non-9-valent HR genotype varied over the study period, similar patterns were observed across multiple age groups rather than being restricted to vaccine-eligible cohorts. Because vaccination history, testing indications, and a stable sampling frame were unavailable, these descriptive findings should not be interpreted as evidence for or against vaccine-associated genotype replacement. Continued assessment of HPV genotype trends remains important for understanding long-term patterns of HPV epidemiology. Consistent with this, previous surveillance studies have demonstrated that HPV genotype distributions may change over time, supporting the need for ongoing monitoring [32]. However, because the number of tested specimens in 2022 was substantially lower than in preceding years due to the availability of only partial-year data, annual fluctuations observed in the present study, particularly in 2022, warrant cautious interpretation.

**Study Strengths.** Taken together, the present findings are broadly consistent with these prior Korean reports. Accordingly, the principal contribution of this study lies not in the discovery of novel genotype or age-related epidemiology, but in the scale and 8-year longitudinal laboratory coverage of the dataset (*n* = 530,808), which enables more stable characterization of less common genotypes and multi-year trend patterns than previously feasible, using standardized genotyping methods consistent across the study period.

**Study Limitations.** Despite these strengths, several limitations should be noted. First, the dataset was specimen-based rather than patient-based. Because analyses relied on laboratory accession numbers without unique patient identifiers, repeat testing from the same individual could not be ruled out. Because HPV-positive or otherwise clinically high-risk women may be more likely to undergo repeat testing than HPV-negative women, repeated specimens could disproportionately influence the observed positivity rates and genotype distributions. Accordingly, the findings reflect specimen-level rather than patient-level observations, and comparisons across age groups should be interpreted descriptively. Temporal trends were similarly described qualitatively based on observed patterns rather than through formal trend testing, consistent with the descriptive nature of the analyses given the specimen-based structure of the dataset.

In addition, individual clinical information—such as vaccination history, cytologic findings, and clinical outcomes—was not available. Because the data were cross-sectional, it was not possible to evaluate patterns of incidence, persistence, or viral clearance over time. Information on anatomical sampling sites was also not available, which may have introduced heterogeneity in specimen type across the cohort. The relative proportion of clinician-referred versus self-requested specimens could not be determined, limiting further characterization of the testing indications underlying this cohort. Finally, because the dataset was derived from clinician referrals and self-requested testing rather than from a structured population-based screening program, individuals at increased risk of HPV infection may have been overrepresented. Accordingly, the reported HPV positivity rates and genotype distributions should be interpreted as specimen-based observations and may not be representative of the general Korean female population.

## 5. Conclusions

This large-scale laboratory-based analysis provides an overview of HPV positivity and genotype distribution among Korean female specimens. HPV positivity showed a U-shaped age pattern, and both HR and LR genotypes were frequently detected across age groups. In addition, 24.4% of specimens were positive for at least one HR genotype not included in the 9-valent vaccine. As this cohort reflects clinically tested rather than population-based specimens, these findings should be interpreted as descriptive epidemiologic observations rather than population prevalence estimates. Future studies incorporating patient-level and clinical outcome data may help further clarify the long-term implications of circulating non-9-valent HR genotypes. These findings contribute to the understanding of HPV genotype distribution among clinically tested Korean females and support continued surveillance of circulating HPV genotypes.

The substantial proportion of non-9-valent HR genotypes detected in this dataset underscores the importance of continued genotype-level surveillance among clinically tested populations, which may support laboratory-based monitoring efforts and inform future public health resource planning. As individual vaccination history, cytologic findings, and clinical outcomes were not available in this dataset, future studies linking genotype-level data with such information would be valuable to further characterize the clinical significance of circulating non-9-valent genotypes in the Korean context. In particular, future vaccine-related arguments should ultimately be grounded in genotype-specific lesion or cancer attribution data rather than raw infection frequency; as such data were unavailable in this cross-sectional dataset, this represents an important direction for future research.

## Figures and Tables

**Table 1:** Annual distribution of HPV genotypes among Korean females (2014–2022).

High-Risk Genotypes										
**HPV Type**	**Total (*n* = 530,808)**	**2014 (*n* = 40,184)**	**2015 (*n* = 51,703)**	**2016 (*n* = 36,946)**	**2017 (*n* = 76,811)**	**2018 (*n* = 79,092)**	**2019 (*n* = 79,887)**	**2020 (*n* = 75,336)**	**2021 (*n* = 82,000)**	**2022 (*n* = 8849)**
16	22,885 (4.3)	1476 (3.7)	2039 (3.9)	1510 (4.1)	3495 (4.6)	3617 (4.6)	3599 (4.5)	3279 (4.4)	3470 (4.2)	400 (4.5)
18	9993 (1.9)	618 (1.5)	989 (1.9)	704 (1.9)	1542 (2.0)	1555 (2.0)	1593 (2.0)	1431 (1.9)	1399 (1.7)	162 (1.8)
26	1277 (0.2)	57 (0.1)	104 (0.2)	94 (0.3)	160 (0.2)	187 (0.2)	195 (0.2)	210 (0.3)	232 (0.3)	38 (0.4)
31	9995 (1.9)	695 (1.7)	984 (1.9)	732 (2.0)	1504 (2.0)	1551 (2.0)	1577 (2.0)	1374 (1.8)	1430 (1.7)	148 (1.7)
33	7094 (1.3)	454 (1.1)	746 (1.4)	494 (1.3)	1150 (1.5)	1074 (1.4)	1088 (1.4)	936 (1.2)	1034 (1.3)	118 (1.3)
35	11,007 (2.1)	667 (1.7)	987 (1.9)	779 (2.1)	1692 (2.2)	1741 (2.2)	1700 (2.1)	1554 (2.1)	1692 (2.1)	195 (2.2)
39	20,308 (3.8)	1169 (2.9)	1582 (3.1)	1196 (3.2)	2781 (3.6)	3254 (4.1)	3474 (4.3)	3120 (4.1)	3314 (4.0)	418 (4.7)
45	5346 (1.0)	267 (0.7)	386 (0.7)	320 (0.9)	716 (0.9)	767 (1.0)	913 (1.1)	910 (1.2)	955 (1.2)	112 (1.3)
51	21,805 (4.1)	1198 (3)	1812 (3.5)	1296 (3.5)	2957 (3.8)	3474 (4.4)	3805 (4.8)	3354 (4.5)	3481 (4.2)	428 (4.8)
52	38,062 (7.2)	2080 (5.2)	3152 (6.1)	2456 (6.6)	5301 (6.9)	5646 (7.1)	6482 (8.1)	5974 (7.9)	6305 (7.7)	666 (7.5)
53	33,265 (6.3)	1982 (4.9)	3007 (5.8)	2353 (6.4)	4960 (6.5)	5150 (6.5)	5318 (6.7)	4739 (6.3)	5158 (6.3)	598 (6.8)
56	19,925 (3.8)	961 (2.4)	1562 (3.0)	1203 (3.3)	2898 (3.8)	3262 (4.1)	3234 (4.0)	3113 (4.1)	3299 (4.0)	393 (4.4)
58	26,817 (5.1)	1526 (3.8)	2426 (4.7)	1994 (5.4)	3972 (5.2)	4125 (5.2)	4276 (5.4)	3837 (5.1)	4159 (5.1)	502 (5.7)
59	10,255 (1.9)	592 (1.5)	931 (1.8)	659 (1.8)	1469 (1.9)	1596 (2.0)	1676 (2.1)	1474 (2.0)	1664 (2.0)	194 (2.2)
66	16,904 (3.2)	943 (2.3)	1499 (2.9)	1185 (3.2)	2562 (3.3)	2629 (3.3)	2588 (3.2)	2484 (3.3)	2711 (3.3)	303 (3.4)
68	25,803 (4.9)	1503 (3.7)	2357 (4.6)	1770 (4.8)	3912 (5.1)	4049 (5.1)	4064 (5.1)	3660 (4.9)	4001 (4.9)	487 (5.5)
69	2150 (0.4)	167 (0.4)	176 (0.3)	175 (0.5)	356 (0.5)	321 (0.4)	304 (0.4)	289 (0.4)	326 (0.4)	36 (0.4)
73	2667 (0.5)	172 (0.4)	244 (0.5)	157 (0.4)	407 (0.5)	416 (0.5)	425 (0.5)	401 (0.5)	395 (0.5)	50 (0.6)
82	7584 (1.4)	458 (1.1)	678 (1.3)	506 (1.4)	1155 (1.5)	1184 (1.5)	1241 (1.6)	1092 (1.4)	1133 (1.4)	137 (1.5)
**Low-Risk Genotypes**										
6	13,266 (2.5)	944 (2.3)	1218 (2.4)	943 (2.6)	1914 (2.5)	2153 (2.7)	2055 (2.6)	1874 (2.5)	1961 (2.4)	204 (2.3)
11	3391 (0.6)	241 (0.6)	330 (0.6)	218 (0.6)	502 (0.7)	540 (0.7)	528 (0.7)	500 (0.7)	487 (0.6)	45 (0.5)
40	12,211 (2.3)	745 (1.9)	1082 (2.1)	854 (2.3)	1772 (2.3)	1799 (2.3)	1964 (2.5)	1840 (2.4)	1937 (2.4)	218 (2.5)
42	18,899 (3.6)	989 (2.5)	1583 (3.1)	1236 (3.3)	2749 (3.6)	2947 (3.7)	3079 (3.9)	2790 (3.7)	3115 (3.8)	411 (4.6)
43	14,483 (2.7)	851 (2.1)	1391 (2.7)	1025 (2.8)	2160 (2.8)	2271 (2.9)	2257 (2.8)	1991 (2.6)	2265 (2.8)	272 (3.1)
44	16,644 (3.1)	916 (2.3)	1465 (2.8)	1153 (3.1)	2460 (3.2)	2525 (3.2)	2650 (3.3)	2529 (3.4)	2634 (3.2)	312 (3.5)
54	28,713 (5.4)	1660 (4.1)	2457 (4.8)	1928 (5.2)	4228 (5.5)	4451 (5.6)	4539 (5.7)	4250 (5.6)	4661 (5.7)	539 (6.1)
61	15,840 (3.0)	978 (2.4)	1483 (2.9)	1177 (3.2)	2401 (3.1)	2422 (3.1)	2409 (3.0)	2084 (2.8)	2565 (3.1)	321 (3.6)
70	21,414 (4.0)	1281 (3.2)	2033 (3.9)	1569 (4.2)	3352 (4.4)	3258 (4.1)	3334 (4.2)	3000 (4.0)	3181 (3.9)	406 (4.6)

Values are described as number (%), representing the total number and percentage of specimens positive for each genotype, including both single- and multiple-type infections. HPV, human papillomavirus.

**Table 2:** HPV genotype multiplicity by year (2014–2022).

Infection Multiplicity	Total Infections	2014 (*n* = 40,184)	2015 (*n* = 51,703)	2016 (*n* = 36,946)	2017 (*n* = 76,811)	2018 (*n* = 79,092)	2019 (*n* = 79,887)	2020 (*n* = 75,336)	2021 (*n* = 82,000)	2022 (*n* = 8849)
1 genotype	128,769	8597 (21.4)	12,347 (23.9)	9177 (24.8)	19,502 (25.4)	19,708 (24.9)	19,484 (24.4)	17,935 (23.8)	19,833 (24.2)	2186 (24.7)
2 genotypes	56,680	3547 (8.8)	5183 (10.0)	3964 (10.7)	8583 (11.2)	8773 (11.1)	8796 (11.0)	8069 (10.7)	8741 (10.7)	1024 (11.6)
3 genotypes	26,216	1523 (3.8)	2351 (4.5)	1841 (5.0)	3813 (5.0)	4150 (5.2)	4178 (5.2)	3767 (5.0)	4096 (5.0)	497 (5.6)
4 genotypes	12,571	662 (1.6)	1050 (2.0)	805 (2.2)	1910 (2.5)	2019 (2.6)	2090 (2.6)	1838 (2.4)	1958 (2.4)	239 (2.7)
5 genotypes	5995	265 (0.7)	479 (0.9)	385 (1.0)	824 (1.1)	940 (1.2)	1051 (1.3)	946 (1.3)	978 (1.2)	127 (1.4)
6 genotypes	2848	105 (0.3)	219 (0.4)	173 (0.5)	385 (0.5)	425 (0.5)	500 (0.6)	509 (0.7)	466 (0.6)	66 (0.7)
7 genotypes	1355	51 (0.1)	89 (0.2)	75 (0.2)	185 (0.2)	203 (0.3)	269 (0.3)	219 (0.3)	234 (0.3)	30 (0.3)
8 genotypes	681	28 (0.1)	37 (0.1)	27 (0.1)	86 (0.1)	113 (0.1)	142 (0.2)	111 (0.1)	122 (0.1)	15 (0.2)
≥9 genotypes	511	15 (0.0)	11 (0.0)	14 (0.0)	39 (0.1)	63 (0.1)	117 (0.1)	117 (0.2)	128 (0.2)	7 (0.1)

Values are described as number (%) or number only. Total infections represent the cumulative counts across study years. Percentages were calculated using the annual number of tested specimens (*n*) as the denominator. HPV, human papillomavirus.

**Table 3:** HPV co-infection profiles by year (2014–2022).

Co-Infection Profile	Total Infections	2014 (*n* = 40,184)	2015 (*n* = 51,703)	2016 (*n* = 36,946)	2017 (*n* = 76,811)	2018 (*n* = 79,092)	2019 (*n* = 79,887)	2020 (*n* = 75,336)	2021 (*n* = 82,000)	2022 (*n* = 8849)
≥2 genotypes	106,857	6196 (15.4)	9419 (18.2)	7284 (19.7)	15,825 (20.6)	16,686 (21.1)	17,143 (21.5)	15,576 (20.7)	16,723 (20.4)	2005 (22.7)
≥2 HR genotypes	66,553	3713 (9.2)	5694 (11.0)	4330 (11.7)	9693 (12.6)	10,450 (13.2)	10,983 (13.7)	9928 (13.2)	10,494 (12.8)	1268 (14.3)
≥2 LR genotypes	26,184	1409 (3.5)	2233 (4.3)	1830 (5.0)	3916 (5.1)	4064 (5.1)	4244 (5.3)	3802 (5.0)	4145(5.1)	541 (6.1)
≥2 HR & ≥2 LR genotypes	12,157	555 (1.4)	976 (1.9)	784 (2.1)	1762 (2.3)	1925 (2.4)	2056 (2.6)	1865 (2.5)	1971 (2.4)	263 (3.0)
1 HR & 1 LR genotype	26,277	1629 (4.1)	2468 (4.8)	1908 (5.2)	3978 (5.2)	4097 (5.2)	3972 (5.0)	3711 (4.9)	4055 (4.9)	459 (5.2)
Concurrent HPV-16/18 infection	938	37 (0.1)	74 (0.1)	40 (0.1)	118 (0.2)	152 (0.2)	173 (0.2)	177 (0.2)	149 (0.2)	18 (0.2)
Concurrent HPV-52/53/58 infection (≥2 genotypes)	10,242	513 (1.3)	815 (1.6)	704 (1.9)	1456 (1.9)	1495 (1.9)	1869 (2.3)	1575 (2.1)	1648 (2.0)	167 (1.9)

Values are described as number (%) or number only. Total infections represent the cumulative counts across study years. Percentages were calculated using the annual number of tested specimens (*n*) as the denominator. The categories “≥2 HR genotypes” and “≥2 LR genotypes” are not mutually exclusive. “≥2 HR & ≥2 LR” represents specimens counted in both categories, whereas “1 HR & 1 LR” represents specimens with exactly one high-risk and one low-risk genotype. These additional categories facilitate reconciliation of the total number of specimens with ≥2 detected genotypes. Concurrent HPV52/53/58 infection was defined as concurrent detection of at least two of HPV52, HPV53, and HPV58. HPV, human papillomavirus.

**Table 4:** Common high-risk HPV genotypes and 9-valent vaccine inclusion status among Korean females (2014–2022).

Category	Genotype(s)	Frequency (%)	IARC Group	Notes
Bivalent vaccine genotypes	16, 18			Cervarix
Quadrivalent vaccine genotypes	6, 11, 16, 18			Gardasil 4
9-valent vaccine genotypes	6, 11, 16, 18, 31, 33, 45, 52, 58			Gardasil 9
	52	7.2	1	Included in 9-valent vaccine
Most frequently detected HR genotypes (overall)	53	6.3	2B	Not included in 9-valent vaccine
	58	5.1	1	Included in 9-valent vaccine
	68	4.9	2A	Not included in 9-valent vaccine
	16	4.3	1	Included in 9-valent vaccine
	51	4.1	1	Not included in 9-valent vaccine
	39	3.8	1	Not included in 9-valent vaccine
	56	3.8	1	Not included in 9-valent vaccine
**≥1 assay-defined non-9-valent HR genotype**	26, 35, 39, 51, 53, 56, 59, 66, 68, 73, 82	129,663 (24.4)	Group 1, 2A, 2B	Anyplex assay-defined HR
**≥1 IARC Group 1 non-vaccine genotype**	35, 39, 51, 56, 59	72,721 (13.7)	Group 1	Non-vaccine carcinogenic types
**≥1 IARC Group 2A genotype**	68	25,803 (4.9)	Group 2A	Probably carcinogenic
**≥1 IARC Group 2B genotype**	26, 53, 66, 73, 82	56,148 (10.6)	Group 2B	Possibly carcinogenic
**≥1 IARC Group 1 or 2A genotype**	35, 39, 51, 56, 59, 68	92,109 (17.4)	Group 1 + 2A	—

Values are presented as number (%) of tested specimens (*n* = 530,808). Percentages were calculated using the total number of tested specimens as the denominator. IARC classification is based on the International Agency for Research on Cancer (IARC) Monograph 100B (2012): Group 1, carcinogenic to humans; Group 2A, probably carcinogenic to humans; Group 2B, possibly carcinogenic to humans. Among the frequently detected non-9-valent HR genotypes, the majority (51, 39, 56) remain classified as Group 1 despite exclusion from the 9-valent vaccine, whereas HPV 53 and 68 carry comparatively weaker carcinogenic evidence (Group 2B and 2A, respectively). HPV, human papillomavirus; HR, high-risk; IARC, International Agency for Research on Cancer.

## Data Availability

The data presented in this study are available on request from the corresponding author due to institutional data-sharing restrictions.

## Supplementary Materials

The following supporting information can be downloaded at: https://www.mdpi.com/article/10.3390/jcm15176853/s1. Supplementary Table S1: Age-group denominators and HPV positivity for age-stratified analyses; Supplementary Table S2: Multiple-type HPV infection by age group and genotype risk classification (2014–2022); Supplementary Table S3: Age-by-calendar year proportion of specimens with ≥1 assay-defined non-9-valent high-risk HPV genotype (2014–2022); Supplementary Table S4: Age-specific proportion of specimens with ≥1 assay-defined non-9-valent high-risk HPV genotype.

## Abbreviations

The following abbreviations are used in this manuscript:

HPV	Human papillomavirus
HR	High-risk
LR	Low-risk
IARC	International Agency for Research on Cancer
NIP	National Immunization Program
PCR	Polymerase chain reaction
