# Supplementary material for: Human Papillomavirus Positivity and Genotype Distribution Among Korean Females: A Large-Scale Laboratory-Based Analysis
Source: J Clin Med. 2026 Sep 4;15(17):6853. doi: 10.3390/jcm15176853 (PMC13566854; doi:10.3390/jcm15176853)
Supplement: Supplementary file 1 [file jcm-15-06853-s001.zip › Supplementary_Table_S1.pdf]

Supplementary Table S1. Age-group denominators and HPV positivity for age-stratified analyses

Age group	Tested specimens (n)	HPV positive, n(%)	HR-HPV positive, n(%)	LR-HPV positive, n(%)
10-19	4,157	2,729(65.6)	2,447(58.9)	1,705(41.0)
20-29	103,280	61,262(59.3)	53,760(52.1)	28,776(27.9)
30-39	148,940	63,013(42.3)	53,922(36.2)	22,936(15.4)
40-49	141,160	51,474(36.5)	42,153(29.9)	19,671(13.9)
50-59	94,248	38,343(40.7)	31,141(33.0)	16,361(17.4)
60-69	30,851	14,632(47.4)	12,542(40.7)	6,210(20.1)
≥70	8,172	4,173(51.1)	3,763(46.0)	1,600(19.6)
**Total**	**530,808**	**235,626(44.4)**	**199,728(37.6)**	**97,259(18.3)**

Values are presented as n (%).

Percentages were calculated using the total number of tested specimens within each age group as the denominator.

Only female specimens with valid age information were included in age-stratified analyses. HR-HPV and LR-HPV positivity were not mutually exclusive.

The denominators reported in this table were used for the age-specific positivity estimates presented in Fig. 2.
