# Supplementary material for: Human Papillomavirus Positivity and Genotype Distribution Among Korean Females: A Large-Scale Laboratory-Based Analysis
Source: J Clin Med. 2026 Sep 4;15(17):6853. doi: 10.3390/jcm15176853 (PMC13566854; doi:10.3390/jcm15176853)
Supplement: Supplementary file 1 [file jcm-15-06853-s001.zip › Supplementary_Table_S2.pdf]

Supplementary Table S2. Multiple-type HPV infection by age group and genotype risk classification (2014–2022)

Age group	Tested specimens (n)	Multiple infections (≥2 genotypes), n(%)	HR-included, n(%)	LR-included, n(%)
10-19	4,157	1,985(47.8)	1,547(37.2)	718(17.3)
20-29	103,280	35,310(34.2)	25,944(25.1)	8,019(7.8)
30-39	148,940	25,847(17.4)	17,680(11.9)	4,082(2.7)
40-49	141,160	18,945(13.4)	11,854(8.4)	3,202(2.3)
50-59	94,248	15,760(16.7)	9,756(10.4)	3,058(3.2)
60-69	30,851	6,935(22.5)	4,738(15.4)	1,366(4.4)
≥70	8,172	2,075(25.4)	1,544(18.9)	394(4.8)
**Total**	**530,808**	**106,857(20.1)**	**73,063(13.8)**	**20,839(3.9)**

Values are described as n (%).

Percentages were calculated using the total number of tested specimens in each age group as the denominator.

HR-included and LR-included denote multiple-type infections in which at least one high-risk or low-risk genotype, respectively, was detected (categories not mutually exclusive).

HPV, human papillomavirus; HR, high-risk; LR, low-risk.
