# Supplementary material for: Human Papillomavirus Positivity and Genotype Distribution Among Korean Females: A Large-Scale Laboratory-Based Analysis
Source: J Clin Med. 2026 Sep 4;15(17):6853. doi: 10.3390/jcm15176853 (PMC13566854; doi:10.3390/jcm15176853)
Supplement: Supplementary file 1 [file jcm-15-06853-s001.zip › Supplementary_Table_S3.pdf]

Supplementary Table S3. Age-by-calendar-year proportion of specimens with ≥1 assay-defined non–9-valent high-risk HPV genotype (2014–2022)

Year	Tested specimens (n)	Specimens with ≥1 assay-defined non–9-valent HR genotype, n(%)	10s (%)	20s (%)	30s (%)	40s (%)	50s (%)	60s (%)	≥70s (%)
2014	40,184	7,739(19.3)	39.1	28.9	16.8	14.9	16.5	20.7	22.0
2015	51,703	11,563(22.4)	41.3	32.5	19.6	18.1	19.0	20.2	24.7
2016	36,946	8,770(23.7)	41.0	35.6	21.6	18.5	21.6	24.7	27.1
2017	76,811	19,351(25.2)	47.7	38.0	22.5	20.0	22.6	28.9	29.5
2018	79,092	20,369(25.8)	49.1	38.7	22.9	20.4	22.7	28.1	30.9
2019	79,887	20,603(25.8)	54.5	40.0	23.6	19.2	22.1	27.8	33.1
2020	75,336	18,626(24.7)	48.1	38.3	22.0	17.9	20.9	27.0	31.6
2021	82,000	20,247(24.7)	49.6	39.0	23.1	16.8	21.0	26.8	29.3
2022*	8,849	2,395(27.1)	52.4	40.8	27.3	18.0	21.0	27.3	31.6
**Overall**	**530,808**	**129,663(24.4)**	**47.2**	**37.1**	**21.9**	**18.5**	**21.3**	**26.7**	**29.7**

Values are presented as n (%).

Percentages in the age-group columns were calculated using the total number of tested specimens within each year-by-age-group cell as the denominator. Overall percentages were calculated using the total number of tested specimens within each age group across the entire study period.

Data for 2022 represent a partial year of specimen collection and should be interpreted with caution.

HPV, human papillomavirus; HR, high-risk.
