# Supplementary material for: Human Papillomavirus Positivity and Genotype Distribution Among Korean Females: A Large-Scale Laboratory-Based Analysis
Source: J Clin Med. 2026 Sep 4;15(17):6853. doi: 10.3390/jcm15176853 (PMC13566854; doi:10.3390/jcm15176853)
Supplement: Supplementary file 1 [file jcm-15-06853-s001.zip › Supplementary_Table_S4.pdf]

Supplementary Table S4. Age-specific proportion of specimens with ≥1 assay-defined non–9-valent high-risk HPV genotype

Age group	Tested specimens (n)	Specimens with ≥1 non–9-valent HR genotype, n(%)
10-19	4,157	1,963(47.2)
20-29	103,280	38,269(37.1)
30-39	148,940	32,651(21.9)
40-49	141,160	26,057(18.5)
50-59	94,248	20,063(21.3)
60-69	30,851	8,229(26.7)
≥70	8,172	2,431(29.7)
**Total**	**530,808**	129,663(24.4)

Values are described as n (%).
Percentages were calculated using the total number of tested specimens in each age group as the denominator.
Non-9-valent HR genotypes were defined as high-risk genotypes not included in the 9-valent vaccine (i.e., excluding HPV16, 18, 31, 33, 45, 52, and 58).
HPV, human papillomavirus; HR, high-risk.
